# Extracellular vesicles transfer chromatin-like structures that induce non-mutational dysfunction of p53 in bone marrow stem cells

**DOI:** 10.1038/s41421-022-00505-z

**Published:** 2023-01-31

**Authors:** Jamal Ghanam, Venkatesh Kumar Chetty, Srishti Anchan, Laura Reetz, Qiqi Yang, Emeline Rideau, Xiaomin Liu, Ingo Lieberwirth, Anna Wrobeln, Peter Hoyer, Dirk Reinhardt, Basant Kumar Thakur

**Affiliations:** 1 Department of Pediatrics III, University Hospital Essen, Essen, Germany; 2 Max Planck Institute for Polymer Research, Mainz, Germany; 3 Laboratory for Molecular Engineering of Optoelectronic Nanomaterials, Institute of Chemical Sciences and Engineering (ISIC), École Polytechnique Fédérale de Lausanne (EPFL), Station 6, Lausanne, Switzerland; 4 Institute of Physiology, University Hospital Essen, Essen, Germany; 5 Department of Pediatrics II, University Hospital Essen, Essen, Germany

**Keywords:** Multivesicular bodies, Acute myeloid leukaemia

Dear Editor,

Small extracellular vesicle (sEV)-DNA has recently emerged as a promising biomarker for cancer diagnosis and prognosis^1^. Despite the growing interest in EV-DNA, many questions related to its nature, loading mechanism, localization, and post-shedding function(s) remain unrevealed. Recently, we have published evidence suggesting an unequal distribution of sEV-DNA between different compartments of the recipient cells, including the nucleus^2^. This finding motivated us to ask whether sEV-DNA is associated with proteins and what is the consequence of this association in the recipient cells. Although histones are abundant in sEVs^3^, whether they are free or associated with sEV-DNA and what is the effect of this association is unknown.

Technically, one of the significant limitations in sEV-DNA-related studies is the use of suitable methods to isolate sEVs devoid of non-EV contaminants, such as cell-free DNA (cfDNA) and apoptotic bodies. To address this issue, we employed the combination of Tangential flow filtration, Size exclusion chromatography, and Ultrafiltration known as TSU (Supplementary Fig. S1a). sEVs were isolated from various cell lines and characterized according to MISEV2018 guidelines (Fig. 1a–d; Supplementary Fig. S1b–e). We have previously shown that TSU provides sEVs that are deficient in cfDNA and apoptotic bodies^2^. Additionally, sEVs were isolated from a pediatric AML cell line (MV4-11) cultured in the presence of EdU to label sEV-DNA and DNase I to digest cfDNA or DNA associated with the outer surface of sEVs (Supplementary Fig. S2a–c). Transfer of these sEVs into bone marrow (BM)-derived mesenchymal stem cells (BM-MSCs) showed that EV-DNA uptake rate was not affected, suggesting that cfDNA or DNA associated with the outer surface of sEVs may not influence EV-DNA uptake (Supplementary Fig. S2d). Further, our results confirmed the presence of histones along with methylated double-stranded DNA (dsDNA) in sEVs (Fig. 1e, f; Supplementary Fig. S3a), as previously reported^4,5^. Using different physical and biochemical methods (Fig. 1g), we have demonstrated that sEVs transfer chromatin-like structures (we termed EV-chromatin), in which many proteins are associated with EV-DNA. With atomic force microscopy (AFM), we documented the presence of EV-chromatin in sEVs derived from MV4-11 (Fig. 1h). Next, we pulled down the sEV-dsDNA and performed non-targeted mass spectrometry to identify the co-precipitated proteins directly interacting with EV-DNA. The data indicate the association of ~30 proteins with EV-DNA, including core histones like H2B and H4, and S100 proteins (Fig. 1i; Supplementary Fig. S3b). The presence of H2B in the pulled-down DNA was further confirmed by western blot against GFP (Supplementary Fig. S3c). This suggests, for the first time, that DNA associated with EVs is directly linked with histones, as the AFM pictures show the existence of chromatin fibers in sEV preparations. Further, confocal images indicate the co-uptake of sEV-DNA and histone H2B, which is identified as an EV-chromatin component (Fig. 1k; Supplementary Fig. S4a–d). Contrariwise, Lázaro-Ibáñez et al.^3^ previously reported the presence of nucleosomal patterns in both low- and high-density sEV subgroups. Nonetheless, they have quantified a number of histones in sEVs without characterizing their direct association with EV-DNA^3^.

**Fig. 1:**
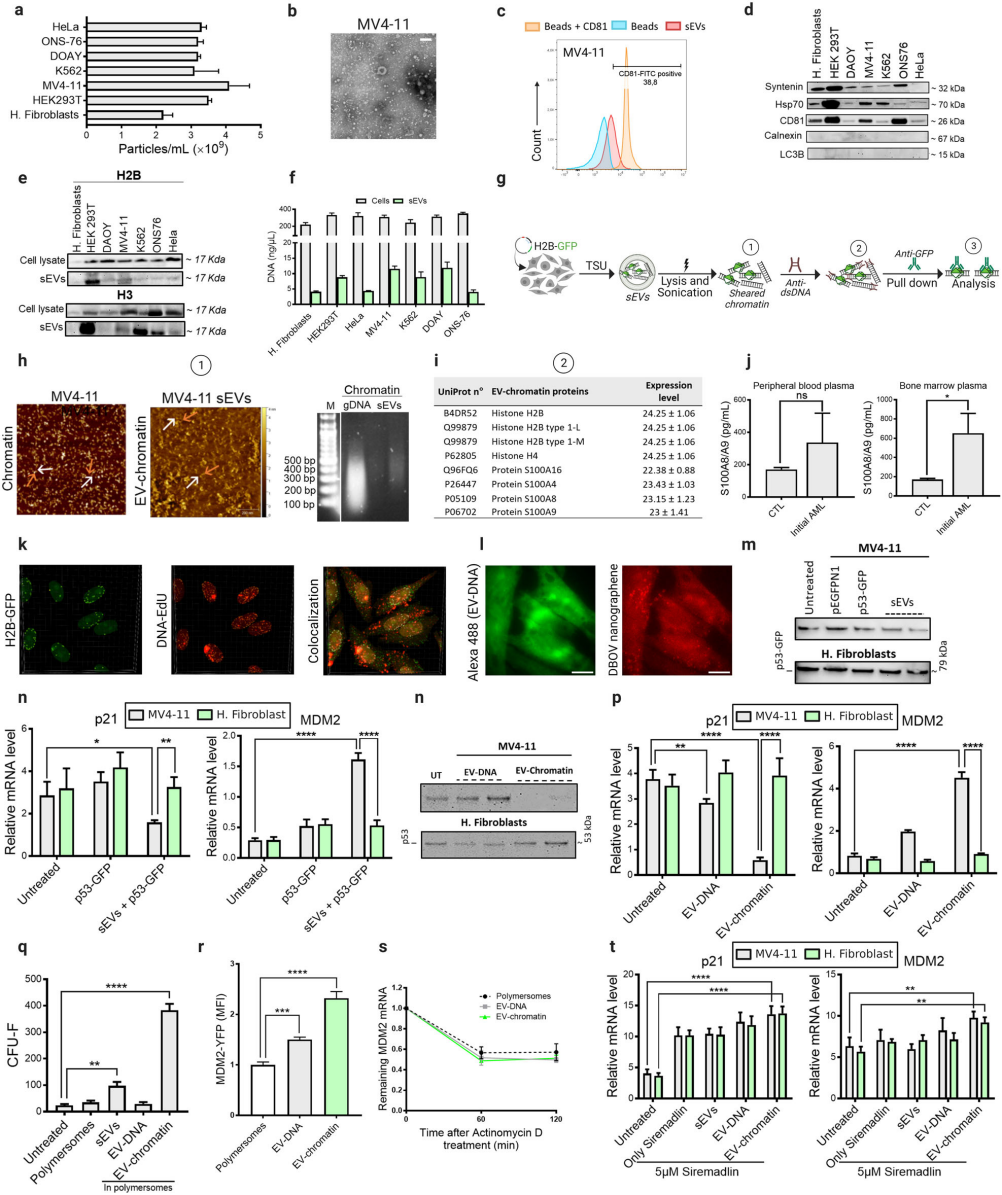
EV-chromatin induces non-mutational dysfunction of p53 in BM stem cells through overexpression of the E3 ligase MDM2. **a** Particle number/mL measured by NTA. **b** TEM negative staining images of MV4-11 sEVs. Scale bar, 0.2 µm. **c** FACS analysis showing the expression of CD81 in MV4-11 sEVs. **d** Western blot analysis with various positive (CD81, HSP70, syntenin) and negative (calnexin and LC3B) markers of sEVs. **e** Expression of H2B and H3 histones in the isolated sEVs. **f** EV-DNA and genomic DNA measured by Quantifluor. **g** Workflow for sEV chromatin characterization. **h** AFM imaging of sheared chromatin highlighting the presence of DNA filaments (white arrows) and protein dots (orange arrows) in both cells and sEV preparations. Chromatin and EV-chromatin showed fragments with sizes ranging from 100 bp to 500 bp after shearing by sonication. **i** Mass spec analysis of the pulled-down chromatin, showing histones (H2B and H4) and S100 as the most abundant proteins of EV-Chromatin. **j** S100A8/A9 levels in sEVs isolated from healthy donors and AML patients. **k** 3D confocal images indicating the co-localized and non-co-localized green (H2B-GFP) and red (EV-DNA) signals in different cell compartments. **l** Characterization of polymersome uptake by BM-MSCs. The fluorescence signals of the green channel (EV-DNA, 488 nm laser) and the red channel (polymersomes, 532 nm laser) were detected inside the BM-MSCs. Scale bars, 10 µm. **m** Western blotting analysis revealing a decrease in p53 level upon treatment with MV4-11 sEVs. **n** MV4-11 sEVs but not fibroblast sEVs downregulate p21 expression in BM-MSCs, which was accompanied by an increase in MDM2 level. **o** p53 degradation upon leukemic EV-chromatin treatment. **p** Relative expression levels of p53 target genes (normalized to the internal control), highlighting the downregulating effect of the leukemic EV-chromatin packaged in polymersomes on p21 compared to fibroblast-derived EV-chromatin. **q** BM-MSC colonies were counted after 7 days of treatment with either sEVs, EV-DNA, or EV-chromatin. **r** Mean YFP fluorescent intensity showing an increase in YFP-tagged MDM2 expression after treatment of BM-MSCs by EV-chromatin and EV-DNA. MFI results were normalized to the untreated cells. **s** *MDM2* mRNA decay in BM-MSCs after Actinomycin D treatment demonstrating comparable mRNA levels in the presence of either EV-chromatin, EV-DNA, or empty polymersomes. **t** Relative expression level of p53 target genes after treatment with 5 µM of Siremadlin. Data were all expressed as means ± SD. *P* value was calculated by one-way or two-way ANOVA. *P < 0.1; **P < 0.01; ***P < 0.001; ****P < 0.0001; ns, not significant.

S100 proteins were highly represented in the protein mixture associated with EV-DNA (Fig. 1i). S100 proteins are calcium-binding proteins that interact with target proteins to trigger many biological processes. These proteins are known to localize in different cell compartments, including the nucleus^6^. This suggests that these proteins likely bind to EV-DNA during or after sEV biogenesis and cargo recruitment (post-biogenesis interaction). S100 proteins interfere with different regulators of cell proliferation and differentiation, including p53, that control the integrity and survival of hematopoietic and mesenchymal stem cells in the BM^7,8^. Like histones, we found that S100 proteins were significantly enriched in sEVs derived from MV4-11 and AML patients compared to those obtained from fibroblast sEVs and healthy donors, respectively (Fig. 1j; Supplementary Fig. S5a).

Following this finding, we attempted to determine whether EV-DNA and EV-chromatin derived from MV4-11 affect the p53 activity of BM-MSCs. Physiologically, hematopoietic and mesenchymal stem cells receive multiple signals from BM stroma, where p53 plays a crucial role in regulating their self-renewal and differentiation rates to support hematopoiesis and prevent tumorigenic transformations^7–9^. p53 stabilizes the transcriptional activation of the cyclin-dependent kinase inhibitor p21 that induces cell cycle arrest required to maintain the balance between proliferation, quiescence, and regeneration via interaction with the BM microenvironment. Our data suggest that AML-sEVs downregulated the expression of p53, and its cell cycle (p21) and apoptosis (BAX and PUMA) target genes in BM-MSCs without affecting their viability (Fig. 1m, n; Supplementary Fig. S5c–f). Interestingly, AML-sEVs upregulated the expression of MDM2 in BM-MSCs (Fig. 1n).

We isolated EV-DNA and prepared EV-chromatin as described in Fig. 1g to investigate whether EV-DNA and/or EV-chromatin are responsible for the p21 inhibition. Next, EV-DNA and EV-chromatin were packaged in engineered polymersomes (Supplementary Fig. S6a, c). We used fluorescence imaging to demonstrate that polymersomes can transport EV-DNA and/or EV-chromatin into cells (Fig. 1l). A similar but more intense effect was observed when we treated BM-MSCs with AML-EV-chromatin packaged in polymersomes (Fig. 1o, p; Supplementary Fig. S6d, e). The proliferation rate of BM-MSCs was accelerated after treatment with EV-chromatin, as shown by the colony-forming unit assay (Fig. 1q; Supplementary Fig. S7a). Furthermore, we found an increase in the expression of YFP, which was tagged to *MDM2* promoter in BM-MSCs treated with EV-chromatin and EV-DNA, whereas the *MDM2* mRNA degradation rate remained the same (Fig. 1r, s), suggesting the possible transcriptional regulation of MDM2 by EV-DNA and EV-chromatin in the nucleus.

It is known that reciprocal interactions between BM-MSCs and AML cells can promote AML progression and resistance to chemotherapy^10^. AML-sEVs play a prominent role in this interaction, as BM stromal and endothelial cells are the preferential BM targets for AML-derived exosomes^11^. Moreover, p53^–/–^ MSCs have shown reduced capacity in supporting normal hematopoiesis due to low secretion of cytokines, such as CXCL12 and CSF1^7^. The transcriptional activity of p53 can be dampened in the presence of wild-type (WT) *TP53* alleles through overexpression of canonical inhibitors such as MDM2. Overexpression of MDM2 has been defined as a mechanism by which cancer cells overcome p53’s tumor suppressive effects, such as AML, in *TP53* WT cancers^12^. Our data suggest that MDM2 mediates complete and partial degradation of p53 in BM-MSCs treated with AML-sEV-chromatin and AML-sEVs, respectively. This indicates that EV-chromatin could be the “active component” when AML-sEVs induce MDM2-mediated degradation of p53 in BM-MSCs. Surprisingly, no significant effects were oberved for DNA derived from AML-sEVs when packaged alone in polymersomes. Most EV-DNA in recipient cells colocalizes with endosomal proteins such as RAB5 and RAB7, indicating its future lysosomal degradation^2^. In contrast, EV-chromatin represents a mixture of DNA and nuclear proteins, such as histones and S100 proteins, which might protect the DNA from cytosolic sensing and degradation. Preventing cytosolic degradation of EV-DNA could be one but not the only role of EV-chromatin proteins. Intriguingly, S100A4 and S100B bind to the tetramerization domain of p53 and disturb the tetramerization of p53 necessary for its nuclear translocation^13^. Conversely, MDM2 reduces p53 acetylation by inhibiting CBP/p300 or recruiting HDAC1 (histone deacetylase 1) to deacetylate p53, favoring p53 ubiquitination, which results in reduced p53 levels^14^. Thus, the EV-chromatin-mediated degradation of p53 could culminate in the synergic activity of S100 proteins that prevent p53 nuclear translocation and MDM2 that mediates its ubiquitination. Nonetheless, the Siremadlin HDM201 inhibition of MDM2 rescued the p53 transcriptional activity (Fig. 1t; Supplementary Fig. S7a), which indicates that both EV-chromatin S100 proteins and MDM2 are required for non-mutational inactivation of p53 (Supplementary Fig. S7b). Similarly, p53 transcriptional activity was rescued after using siRNA to induce *MDM2* gene silencing (Supplementary Fig. S8a–c).

How AML-sEVs interfere with BM-MSCs and other stromal cells to transform BM microenvironment into a leukemic niche is still under investigation. We provided proof of the principle of the existence of chromatin-like structures (EV-chromatin) in AML-sEVs, and we have shown that EV-chromatin could communicate with BM-MSCs and modulate their behavior. We identified non-mutational p53 inactivation involving MDM2 and potentially S100 proteins (associated with EV-chromatin) as a mechanism by which AML-EV-chromatin regulates the proliferation of BM-MSCs. Conversely, we re-established the expression of p53 targets in BM-MSCs by inhibiting MDM2 or *MDM2* gene silencing. Our finding delineates a new mechanism of crosstalk between AML and stromal cells via EV-chromatin, which could be one of the reasons for hematopoietic failure during or after AML therapy. Our data emphasized the importance of targeting the interaction between MDM2 and p53 as a promising treatment strategy in *TP53* WT or functional p53 cancers. However, additional basic and clinical investigations are needed to further elucidate how sEVs and EV-chromatin or “Exogenotin” modulate the molecular pathway mediated by the p53-MDM2 axis in the BM niche.

## Supplementary information

Supplementary information
