# Supplementary material for: Extracellular vesicles transfer chromatin-like structures that induce non-mutational dysfunction of p53 in bone marrow stem cells
Source: Cell Discov. 2023 Jan 31;9:12. doi: 10.1038/s41421-022-00505-z (PMC9887011; doi:10.1038/s41421-022-00505-z)
Supplement: Supplementary file 1 — Supplementary information [file 41421_2022_505_MOESM1_ESM.pdf]

Supplementary information for

Extracellular vesicles transfer chromatin-like structures that induce non-mutational dysfunction of p53 in bone marrow stem cells

Authors: Jamal Ghanam[1], Venkatesh Kumar Chetty[1], Srishti Anchan[1], Laura Reetz[1], Qiqi Yang[2], Emeline Rideau[3], Xiaomin Liu[2], Ingo Lieberwirth[2], Anna Wrobeln[4] , Peter Hoyer[5], Dirk Reinhardt[1], Basant Kumar Thakur[1*]

*Correspondance to: basant-kumar.thakur@uk-essen.de

This file includes:

Materials and Methods
Supplementary References
Figures S1 to S8
Tables S1 and S2

Materials and Methods

Cell culture

Human bone marrow mesenchymal stem cells BM-MSCs (ATCC PCS-500-012) were obtained from American Type Culture Collection and maintained in the Mesenchymal Stem Cell Basal Medium (ATCC PCS-500-030) supplemented with the one Mesenchymal Stem Cell Growth (ATCC PCS-500-041) according to the manufacturer instructions. HeLa (cervical cancer adenocarcinoma), MV4-11 (acute monocytic leukemia), and K562 (Chronic Myelogenous Leukemia Cml) Cell lines were maintained in Roswell Park Memorial Institute 1640 (RPMI1640; Gibco® Life Technologies Corp., USA) with 10% fetal bovine serum and 1% penicillin/streptomycin. HEK293T-CD63-GFP (human embryonic kidney with a GFP tag on CD63) and HEK293T (Transformed human embryonic kidney) cell lines were cultivated and maintained in Dulbecco's minimal essential media without pyruvate (DMEM; Gibco® Life Technologies Corp., USA) with 10% fetal bovine serum (Biowest, France) and 1% penicillin/streptomycin (Gibco® Life Technologies Corp., USA). DAOY (Desmoplastic Cerebellar Medulloblastoma), OSN-76 (Medulloblastoma) cell lines were cultivated and maintained in Dulbecco's minimal essential media (DMEM; Gibco® Life Technologies Corp., USA) with 10% fetal bovine serum (Biowest, France) and 1% penicillin/streptomycin (Gibco® Life Technologies Corp., USA).

Small extracellular vesicles isolation from conditioned media

One batch (8 x 20 mL) of conditioned culture media (CCM) was used to isolate sEVs. Washed cells were cultured for 72 h in 20 mL of the corresponding media containing 10% EV-depleted FBS, which was obtained by ultracentrifugation at 100 000 g (Beckmann Coulter, California, USA) for 18 h and filtration through a 0.22 µm filter. First, the media was subjected to a centrifugation step of 500 g for 10 min to remove cells, and the supernatant was spun again at 3000 g for 20 min to get rid of cell debris and apoptotic bodies and subsequently frozen at -80°C. The pre-cleared media was then filtrated (0.2 µm) and concentrated (to 10 mL) using a TFF-Easy - tangential flow filtration column (HansaBioMed, Estonia). Concentrated media was then loaded onto the pre-flushed (with one volume of 0.2 µm filtrated DPBS) size exclusion chromatography (SEC) column (IZON Science, USA) and voided with 10 mL of 0.2 µm filtrated DPBS. Afterward, sEVs fractions (5 mL) were immediately collected, as

we have previously described [1]. sEV fractions were then concentrated (to 0.5 mL) using Amicon® Ultra-4 Centrifugal filter unit with Ultracel-10 membrane (MWCO = 10kDa; Merck Millipore, Billerica, MA). sEVs were stored at -80 until further use.

To isolate sEVs from DNase I containing supernatants, cells were cultivated uder the same conditions as described above in the presence of 1 ug/mL of cell culture grade DNase I (Roche, Germany).

Isolating sEVs with EdU labled EV-DNA

For EV-DNA-based functional and uptake studies, 5 µM of 5-ethynyl-2'-deoxyuridine (EdU; Thermofisher Scientific, Germany) solution was added to the CCM few hours after seeding the cells (i.e., when cells are attached to the dish-es). EdU is a thymidine analog incorporated into newly synthesized DNA during active DNA replication; thereby, cells treated with EdU release sEVs in which DNA is metabolically labeled with EdU.

Patient samples

Plasma samples (peripheral blood or bone marrow plasma) were collected from patients initially diagnosed with pediatric acute myeloid leukemia under the University Hospital Essen Department of Pediatrics III, AML-BFM Reference Lab. Four peripheral blood plasma and four bone marrow plasma samples were used to isolate sEVs. Appropriate informed oral and written consent was obtained from patients and healthy donors before sample collection under the research protocol approved by the ethics committee of the Medical Faculty, University Hospital of Duisburg-Essen (16-7069-BO).

Extracellular vesicles isolation from patient samples

Samples were first centrifuged for 10 min at 500 g and 4°C to remove red cells. Plasma samples (2 mL) were collected in new tubes, centrifuged at 3000 g and 4°C for 20 min, and the supernatants were stored at -80°C until use. Samples were concentrated to 500 µL in Amicon (R) Ultra - 2ml centrifugal filters (Merck Millipore, Billerica, MA) at 4000 x g. Samples were washed twice with PBS onto the filter to reduce the protein content and prevent clogging. Samples were then loaded onto a qEV2 column (IZON Science, USA). sEVs' fractions were then concentrated using Amicon® Ultra-4 Centrifugal filter unit with Ultracel-10 membrane (Merck Millipore, Billerica, MA) and stored at -80 until further use.

EV-DNA characterization

Genomic DNA and EV-DNA extraction- gDNA and EV-DNA were extracted using the QIAmp DNA kit and QIAmp DNA micro kit, according to the manufacturer's instructions (QIAGEN, Germany). After isolation, EV-DNA samples were eluted in 22 µL nuclease-free water. All DNA samples were stored at −20°C. dsDNA quantification was performed using the sensitive QuantiFluor® ONE dsDNA System (Promega, Germany), providing a fluorescent double-stranded DNA-binding dye (504nmEx/531nmEm).

Characterization of AML-EV-DNA after treatment of MV4-11 cells by DNase I during sEVs biogenesis- EV-DNA was extracted from CCM and sEVs (Fig. S2a) and loaded onto a 1.5% agarose gel (Sigma Aldrich, Germany) and run for 75 min. DNA was then detected using highly sensitive SYBR Gold nucleic acid staining for 30 min at room temperature (Thermo Fisher Scientific, Germany).

Global DNA methylation

The global methylation profile of EV-DNA and gDNA was determined using the MethylFlash™ Global DNA Methylation (5-mC) ELISA Easy Kit (EpigenTek, Brooklyn, NY, USA) according to the manufacturer's instructions. The absorbance at 450 nm was assayed using a Tecan Infinit 200 Microplate Photometer (Tecan, Switzerland). Results were reported as a percentage (%) of 5-mC methylated DNA relative to the input DNA quantity, according to the following formula:

$$5\text{-mC}\% = ((\text{Sample OD} - \text{Negativ control OD}))/(\text{Slope} \times \text{Input DNA}) \times 100$$

Immunoblotting

Total cells and sEV proteins were determined using BCA and micro BCA assay kits (Invitrogen). For sEVs characterization, fractions of 100 µL were first concentrated up to ten times using Amicon® Ultra-4 Centrifugal filter unit with Ultracel-10 membrane (MWCO = 10kDa; Merck Millipore, Billerica, MA). Cell lysates were prepared by digesting cell pellets in RIPA buffer (Thermofisher Scientific). Concentrated vesicle suspensions and cell lysates were then treated with 4X Laemmli buffer (Biorad, Germany) in the presence of beta-Mercaptoethanol and protease and phosphatase inhibitors cocktail at 95°C for 10 min. Samples were then loaded on NuPAGE 4-12 % Gel (Invitrogen, Germany), resolved for 2h (100 V), transferred onto the Immuno-blot PVDF membrane (Merck Millipore), and

subsequently blocked with 5% dry milk (Roth, Germany) in TBS-T (Tris Buffered Saline with 0.1% Tween-20) for 1 hour. The membranes were then incubated overnight at 4°C with the primary antibodies listed in Supplementary Table 1. The blots were vigorously washed whit TBS-T and then incubated with the corresponding secondary antibodies for 90 min at room temperature. Membranes were detected with Pierce ECL plus Western blotting substrate (Thermo Fisher Scientific), and images were taken on Fusion FX Machine (Vilber Lourmat Deutschland GmbH).

Nanoparticle tracking analysis

The Particle's size and concentration were determined by Nanoparticle tracking analysis (NTA) analysis using Nanosight LM10 instrument (Particle Metrix, Germany) equipped with NTA 2.0 analysis software. sEV fractions were diluted (1:100) and then analyzed according to the following conditions: positions- 11, cycles- 5, minimum size- 5nm, maximum size - 150nm, trace length- 15secs, sensitivity- 75%, shutter speed- 75msecs and frame rate- 30.

Transmission electron microscopy

Negative staining was performed at the Electron Microscopy Unit (EMU) of the Imaging Center Essen (IMCES) for sEVs. In addition, FBS18 EV was included as a negative control. Briefly, 3 µl of sEVs were added onto a Formvar-coated 200 mesh copper grid (#SF162, PLANO GmbH) which had a hydrophilic surface due to being exposed to glow discharging for 1.5 minutes (easiGlow™, PELCO). Samples were then negatively stained with 10 µLof 1.5% v/v Phosphotungstic acid (PTA) for 2 min. Excess liquid was removed, and the grids were allowed to dry for at least 2 minutes. Samples were observed using a JEOL JEM-1400 Plus TEM (JEOL) at 120 kV, and the images were processed using ImageJ to determine the average diameter.

Bead-assisted flow cytometry

sEVs were analyzed by flow cytometry for semi-quantitative detection of sEV protein CD81 according to the protocol we described before [1]. Briefly, sEVs were incubated with aldehyde-sulfate latex beads (4 µM; Invitrogen) for 30 min at RT. After removing the unbound beads, 5% BSA was added for blocking for 30 min at RT. Blocked sSEVs-Beads were then stained with CD81-FITC (Beckman

Coulter, Marseille, France). Data were acquired in conventional flow cytometers (BD FACS Aria, BD Biosciences, Heidelberg, Germany) and analyzed using FlowJo™ v10.8 Software (BD Life Sciences).

Chromatin Immuno-precipitation followed by deep sequencing (ChIP-Seq)

ChIP assay was performed using the ab500 chromatin immunoprecipitation kit (Abcam Biotechnology, MA, USA). Cells were seeded onto T175 flasks according to the doubling time. Cells were then scraped and washed twice with DPBS, and aliquots of 1×10^7 were considered for the next ChIP steps. Cells and sEVs (100 µL) were cross-linked in 1% of formaldehyde (ThermoFisher, Germany) for 10 min at room temperature and subsequently neutralized with glycine. Cells and EVs were lysed in lysis buffer containing protease inhibitor (Abcam Biotechnology, MA, USA). Only 100 µL of lysis buffer was used for sEVs, and 4 µL of the protease inhibitor was added on the top. Cells and EVs preparations were sonicated at 4°C (eight and four cycles for cells and EVs, respectively) to shear the chromatin to 200-1000 bp fragments using UP400S Bioruptor (Hielscher, Germany). Sonicated lysates were centrifuged for 10 min at 12 000 rpm at 4°C and supernatants were transferred to new tubes. To check the DNA fragment length, 20 µL of sonicated cells and EVs chromatins were mixed with 100 µL of PCR-grade water (ThermoFisher, Germany), 100 µL of DNA purifying slurry (Abcam Biotechnology, MA, USA), and 1 µL of proteinase K for 30 min at 55°C. 10 µL were then loaded onto a 1.5% agarose gel (Sigma Aldrich), run for 75 min, and detected using SYBR Gold nucleic acid staining for 30 min (Thermo Fisher Scientific, Germany).

Sheared chromatins were then incubated with ChIP grade rabbit polyclonal anti-dsDNA antibody (Abcam Biotechnology, MA, USA) overnight at 4°C on a rotating wheel. The antibody-chromatin mixture was incubated with Protein A beads for 1 h at 4°C, and DNA was purified using the abcam DNA slurry as described before.

ChIP Mass spectrometry and data analysis

Sample preparation, LC-MS/MS, data processing, and data analysis were performed at the EMBL Proteomics Core Facility (Heidelberg, Germany) according to the following protocol:

Sample preparation- Reduction of disulphide bridges in cysteine containing proteins was performed with dithiothreitol (56°C, 30 min, 10 mM in 50 mM HEPES, pH 8.5). Reduced cysteines were

alkylated with 2-chloroacetamide (room temperature, in the dark, 30 min, 20 mM in 50 mM HEPES, pH 8.5). Samples were prepared using the SP3 protocol [2,3] and trypsin (sequencing grade, Promega) was added in an enzyme to protein ratio 1:50 for overnight digestion at 37°C. Next day, peptide recovery in HEPES buffer by collecting supernatant on magnet and combining with second elution wash of beads with HEPES buffer. Peptides were further cleaned up using an OASIS® HLB µElution Plate (Waters) according to manufacturer's instructions.

LC-MS/MS- An UltiMate 3000 RSLC nano LC system (Dionex) fitted with a trapping cartridge (µ-Precolumn C18 PepMap 100, 5µm, 300 µm i.d. x 5 mm, 100 Å) and an analytical column (nanoEase™ M/Z HSS T3 column 75 µm x 250 mm C18, 1.8 µm, 100 Å, Waters). The outlet of the analytical column was coupled directly to an Orbitrap Fusion™ Lumos™ Tribrid™ Mass Spectrometer (Thermo) using the Nanospray Flex™ ion source in positive ion mode.

The peptides were introduced into the Orbitrap Fusion Lumos via a Pico-Tip Emitter 360 µm OD x 20 µm ID; 10 µm tip (CoAnn Technologies) and an applied spray voltage of 2.4 kV, instrument was operated in positive mode. The capillary temperature was set at 275°C. Full mass scans were acquired for a mass range 375-1200 m/z in profile mode in the orbitrap with resolution of 120000. The filling time was set to a maximum of 50 ms, the AGC target was set to standard. The instrument was operated in data dependent acquisition (DDA) mode and MSMS scans were acquired in the Orbitrap with a resolution of 15000, with a fill time of up to 54 ms and a limitation of 2e5 ions (AGC target). A normalized collision energy of 34 was applied. MS2 data was acquired in profile mode.

Data processing, MaxQuant- The raw mass spectrometry data was processed with MaxQuant (v1.6.3.4) [4] and searched against Homo sapiens proteome database (UP000005640) containing common contaminants. The data was searched with the following modifications: Carbamidomethyl (C) (fixed modification), Acetyl (N-term) and Oxidation (M) (variable modifications). A maximum of two missed cleavages was allowed. For protein identification a minimum of 2 unique peptides with a peptide length of at least seven amino acids and a false discovery rate below 0.01 were required on the peptide and protein level. Quantification was performed using iBAQ values [5].

Data analysis- The raw output file of MaxQuant (ProteinGroups.txt – file) was processed using the R programming language (ISBN 3-900051-07-0). As a quality filter, only proteins were allowed that were quantified with at least two unique peptides. Raw iBAQ values were used without normalization. Differential expression was evaluated by computing the respective ratio of raw iBAQ values. In order to try to annotate the ratio (coming from a single replicate) with a p-value, the ratio distribution was assumed to come from a student's t-distribution from which p-values were estimated using the 'pt' function from R. The degrees of freedom were simplified by the number of observed proteins. The false discovery rates calculated from the p-values using the 'p.adjust' function from R. This method was used to get a quick approximation.

Atomic force microscopy

To study DNA-protein association, sheared genomic chromatin and EV-chromatin were analyzed by AFM using Dimension Icon equipped with ScanAsyst FastScann head (Bruker, Germany). Samples were diluted (1:5) in 5 mM Tris pH 8, 12 mM MgCl2, 1 mM EDTA, 5 mM NaCl. 10 µL of diluted samples were dropped on a freshly cleaved mica surface and processed under the following conditions : Experiment: PeakForce QNM in liquid; Cantilever Type: FASTSCAN-C; Resonant Freq.: 300 kHz; Spring Constant: 0.8 N/m; Back side coating: reflective aluminum. The average roughness was calculated using the Gwyddion software to remove the noise and apply the Median Filter on the images as a non-linear digital filtering technique.

Cryo-EM analysis

For cryo-EM examination, samples were vitrified using a Vitrobot Mark V (Thermo Fisher, Hilsboro Oregon) plunging device. 3 µL of the sample dispersion was applied to a Quantifoil or a lacey carbon coated TEM grid that had been glow discharged in an oxygen plasma cleaner (Diener Nano®, Diener electronic, Germany) shortly before. After removing the excess sample solution with filter paper, the grid is immediately plunged into liquid ethane. The specimen is transferred to a TEM (FEI Titan Krios G4) for the subsequent examination, keeping cryogenic conditions. Conventional TEM imaging was done using an acceleration voltage of 300 kV. Micrographs were acquired with a 4k Direct Electron Detection Camera (Gatan K3) under low-dose conditions.

Packaging EV-DNA and EV-chromatin in polymersomes

The polymersomes preparation was carried out as follows. The block copolymer polybutadiene-b-poly(ethylene ethyl phosphate) (PB-b-PEEP) was prepared as described previously [6]. For the blank polymersomes, 20 µL of a (PB(1,4)73-b-PEEP12) solution in CHCl3 (4 mg/mL) was added to a 2 mL glass vial and concentrated in a desiccator under reduced pressure until the solvent was evaporated. An invisible thin film of the neat polymer was thus obtained. Next, 200 µL of PBS was quickly added, and the reaction was left to stir overnight (1250 min-1, 30h) vigorously. The vesicles were prepared as described above for the encapsulation experiments, adding EV-DNA or EV-chromatin to the PBS solution. The prepared vesicles were stored in a refrigerator at 4 °C until the subsequent use.

Treatment of BM-MSCs with sEVs and polymersomes containing EV-DNA or EV-chromatin

For sEVs treatment, BM-MSCs were transiently transfected with p53-wt Cds in pEGPF-N1 vector (Clontech Laboratories, Mountain View, CA, USA) using Lipofectamine 3000 and P3000 reagent according to the manufacturer's instructions (Thermofisher, Germany). Equal numbers of sEVs at an approximately 50:1 ratio (sEVs/recipient cells) were resuspended in FBS18 culture media and added to the transfected BM-MSCs in culture. Cells were incubated for 48h before being scraped and prepared for qRT-PCR or Western blot experiments. For the treatment with EV-DNA and EV-chromatin packaged in polymersomes, attached BM-MSCs cells have received equal amounts of polymersomes calculated according to the DNA concentration.

Studying of EV-DNA uptake in HeLa cells and BM-MSCs

EV-DNA and histone H2B co-uptake by HeLa cells- to generate GFP tagged H2B, HEK293T cells were transiently transfected with H2B-GFP plasmid (Addgene plasmid # 20972 ; http://n2t.net/addgene:20972 ; RRID:Addgene_20972) using Lipofectamine RNAiMAX reagent according to the manufacturer's instructions (Thermofisher, Germany) in Opti-MEM media (Gibco® Life Technologies Corp., USA). Transfected HEK293T cells were then incubated for sEVs isolation in the presence of EdU as described above. After isolation from HEK293T supernatant, sEVs were

resuspended in FBS18 culture media and added to HeLa cells in culture at approximately 50:1 ratio (sEVs/recipient cells).

Cells were fixed and permeabilized as we have described before [1]. EdU click-it reaction was carried out using Click-iT™ EdU Alexa Fluor 647 Imaging kit (ThermoFisher, Germany) following the manufacturer's instructions. Images were acquired in confocal microscopy (Leica TCS SP8) in the corresponding channels (blue- DAPI, green- H2B-GFP, and red- EdU). ImageJ was used to quantify red and green mean fluorescence intensity.

EV-DNA uptake by BM-MSCs- After sEVs isolation from MV4-11 supernatants, sEVs were resuspended in FBS18 culture media and added to BM-MSCs in culture at approximately 50:1 ratio (sEVs/recipient cells). Cells fixation and permeabilization, as well as the click reaction, were performed as we have previously described.

Transcription inhibition by Actinomycin D

For transcription inhibition, BM-MSCs were first incubated with EV-DNA or EV-chromatin for 24h and then treated with 10 µg/mL of Actinomycin D (Sigmaaldrich, Germany) for 0, 1, and 2 h. Cells were then lysed for RNA extraction and the inhibition of transcription was determined with RT-qPCR.

MDM2 reporter assay

To evaluate the ability of AML EV-DNA and EV-chromatin to activate MDM2 promoter, BM-MSCs were transiently transfected with MDM2p-Mdm2-YFP from Uri Alon & Galit Lahav [7] (Addgene plasmid # 53962 ; http://n2t.net/addgene:53962 ; RRID:Addgene_53962) using Lipofectamine RNAiMAX reagent according to the manufacturer's instructions (Thermofisher, Germany). Transfected BM-MSCs cells were then incubated with AML EV-DNA and EV-chromatin for 24h and analyzed by flow cytometry. YFP Data were acquired in conventional flow cytometers (BD FACS Aria, BD Biosciences, Heidelberg, Germany) and analyzed using FlowJo™ v10.8 Software (BD Life Sciences).

MDM2 inhibition

Siremadlin treatment- Siremadlin HDM201 was synthesized by Global Discovery Chemistry at Novartis. For *in vitro* treatment, 2×10^5 BM-MSCs were first cultured in the presence of 5 µM Siremadlin. After 24h, the media was replaced FBS18 media with MV4-11 sEVs, EV-DNA, or EV-chromatin for 24h.

siARN against MDM2- For siRNA treatment, 2×10^5 BM-MSCs were first cultured overnight. After 24h, the media was replaced FBS18 media with MV4-11 sEVs, EV-DNA, or EV-chromatin for 24h. Cells were then transfected with 50 nM of siRNA targeting MDM2 (Thermofisher, Germany) using Lipofectamine RNAiMAX Transfection Reagent (Thermofisher, Germany) in Opti-MEM™ medium. Scrambled siRNA was used as a negative control. The knockdown efficiency was assessed by western blotting.

Colony forming unit assay

For colony-forming unit assays, 100 BM-MSCs were seeded in MethoCultTM GF M3434 (Stem Cell Technologies, Canada) with or without sEVs, EV-DNA, and EV-chromatin. Colonies number was recorded at day 7 after plating.

Reverse transcription-quantitative polymerase chain reaction (RT-qPCR)

BM-MSCs were lysed, and total RNA was extracted using the RNeasy Mini Kit (Qiagen, Germany) according to the manufacturer's instructions. RNA concentration was determined by Nanodrop 1000 Spectrophotometer (Thermofisher, Germany). RNA was reversely transcribed using the Transcriptor First Strand cDNA Synthesis Kit (Roche Applied Science). PCR was performed using FastStart Universal SYBR Green master mix (Rox) (Roche Applied Science) following the manufacturer's protocols. The reaction was done in StepOnePlus™ Real-Time PCR System (ThermoFisher, Germany). The results were calculated using ΔΔCt method and normalized to GAPDH. All the measurements were performed in triplicate and repeated for at least three independent sEVs, EV-DNA, and EV-chromatin preparations. Primer sequences are listed in Supplementary Table 2.

Cell viability

Annexin V-FITC Apoptosis Staining- BM-MSCs treated with either leukemic sEVs, EV-DNA, or EV-chromatin were analyzed using an Annexin V-fluorescein isothiocyanate (FITC)/propidium iodide (PI) Apoptosis Staining Kit according to the manufacturer instructions (Abcam Biotechnology, MA, USA). Cells were resuspended in 500 µl binding buffer, mixed with 5 µl Annexin V-FITC followed by 5 µl PI, and incubated in the dark for 10 min at room temperature. A total of 1×106 BM-MSCs from each group were collected after 48 h of treatment. Data were acquired in conventional flow cytometers (BD

FACS Aria, BD Biosciences, Germany), and results were analyzed using FlowJo™ v10.8 Software (BD Life Sciences, Germany).

MTT assay- BM-MSCs and MV4-11 cell viability was assessed by the reduction 3-(4,5-dimethylthiazol-2-yl)-2,5-diphenyltetrazolium bromide (MTT) (Sigmaaldrich, Germany) measured at 540 nm using a Tecan Infinit 200 Microplate Photometer (Tecan, Switzerland).

Statistical analysis and reproducibility

Data were statistically analyzed using GraphPad Prism 7.0 (GraphPad Software, San Diego, California, USA). Two-tailed Student's t-test analyzed comparisons between two groups, and multiple comparisons were performed by one-way or two-way analysis of variance (ANOVA). All results represent at least three times independent experiments. The data in the figures are expressed as the mean ± standard deviation (SD). Data that found to be statistically significant were represented in the graphs as * for $p < 0.0332$, ** for $p < 0.021$, *** for $p < 0.0002$ and **** for $p < 0.0001$.

Supplementary references

1. Chetty, V. K. *et al.* Efficient Small Extracellular Vesicles (EV) Isolation Method and Evaluation of EV-Associated DNA Role in Cell–Cell Communication in Cancer. *Cancers (Basel).* **14**, (2022).
2. Hughes, C. S. *et al.* Single-pot, solid-phase-enhanced sample preparation for proteomics experiments. *Nat. Protoc.* **14**, 68–85 (2019).
3. Hughes, C. S. *et al.* Ultrasensitive proteome analysis using paramagnetic bead technology. *Mol. Syst. Biol.* **10**, 757 (2014).
4. Cox, J. & Mann, M. MaxQuant enables high peptide identification rates, individualized p.p.b.-range mass accuracies and proteome-wide protein quantification. *Nat. Biotechnol.* **26**, 1367–1372 (2008).
5. Schwanhäusser, B. *et al.* Global quantification of mammalian gene expression control. *Nature* **473**, 337–342 (2011).
6. Rideau, E., Wurm, F. R. & Landfester, K. Giant polymersomes from non-assisted film hydration of phosphate-based block copolymers. *Polym. Chem.* **9**, 5385–5394 (2018).
7. Lahav, G. *et al.* Dynamics of the p53-Mdm2 feedback loop in individual cells. *Nat. Genet.* **36**,

147–150 (2004).

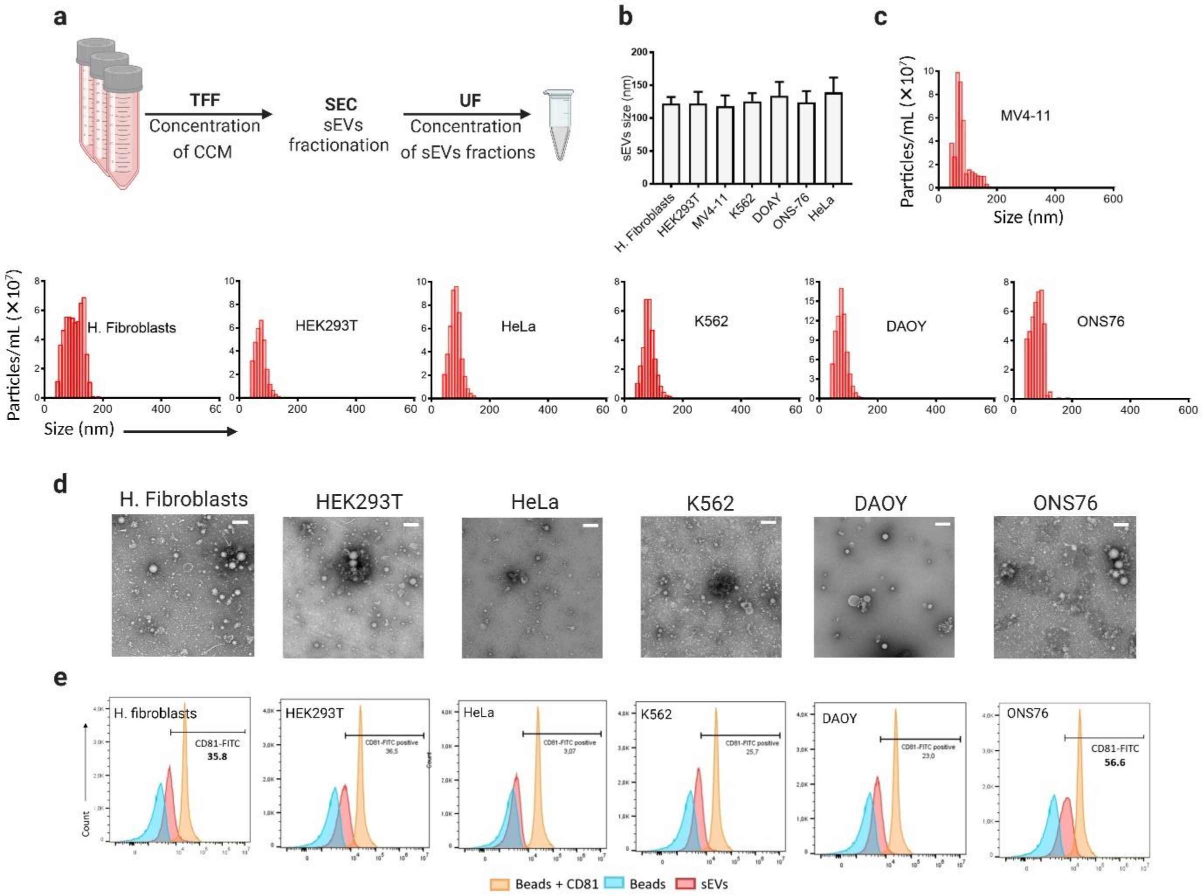

Supplementary Fig. S1. Characterization of cancer and non-cancer small EVs. **a**, Overview of sEVs isolation procedure from CCM. **b**, sEVs size in nm, measured by NTA. Data are shown as mean ± SD. (**c**) sEVs distribution according to the size, determined by NTA. **d**, Imaging of TEM negative staining of sEVs. Scale bars- 0.2 µm. **e**, Flow cytometry analysis showing the expression of CD81 on the surface of the isolated sEVs.

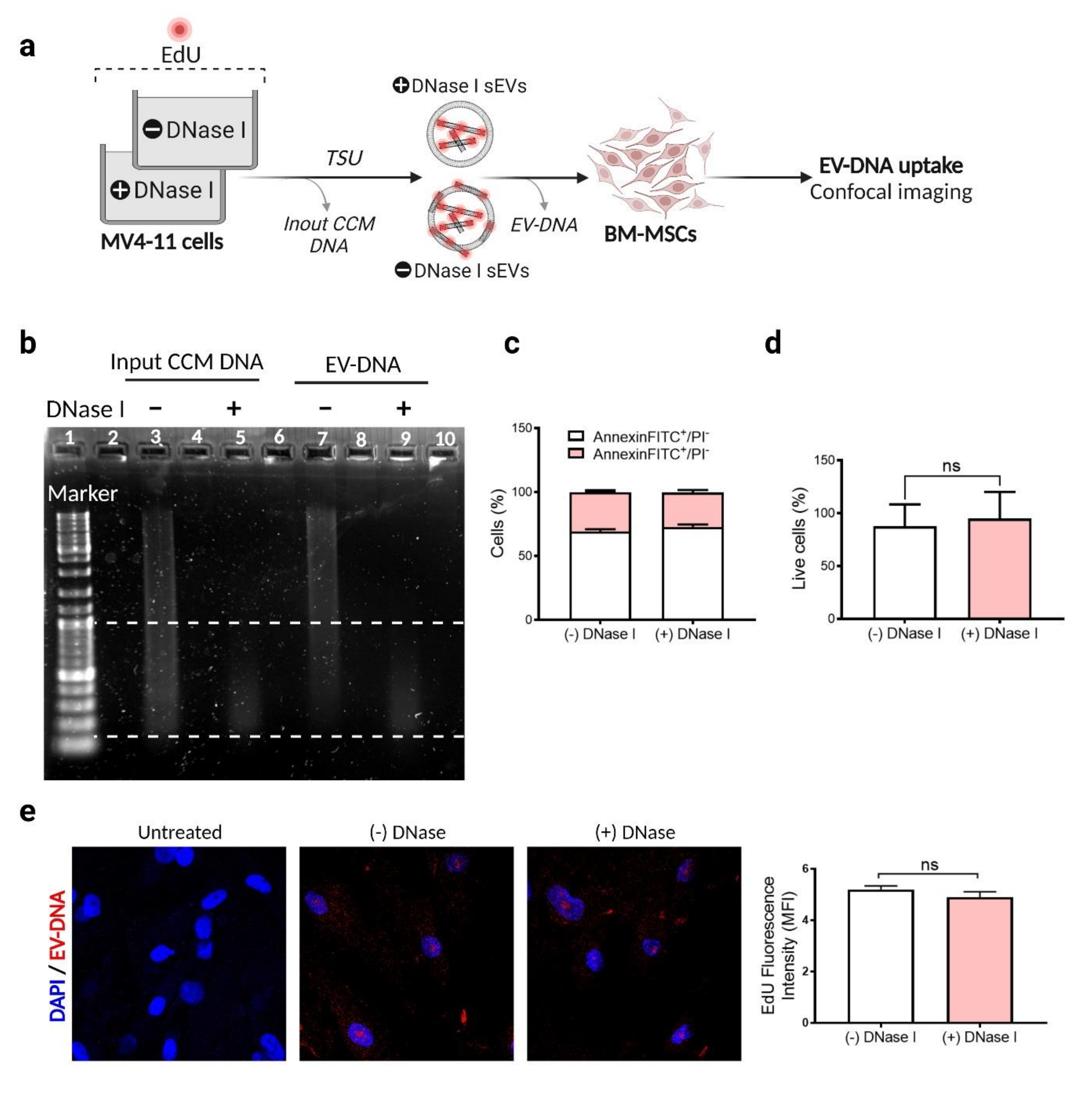

Supplementary Fig. S2. Characterization of AML-EV-DNA uptake by BM-MSCs after treatment of MV4-11 cells by DNase I. **a**, Working flow. **b**, SYBR™ Gold based detection of DNA extracted from TFF-concentrated CCM from MV4-11 cells cultivated without (lane 3) and with (lane 5) DNase I, and sEVs from MV4-11 cells cultivated without (lane 7) and with (lane 9) DNase I. DNA ladder loaded in lane 1 as control for MW size in base pairs. The results are representative of three experiments performed independently. Lanes 2, 4, 8, and 10 are empty. **c**, Apoptosis assay and **d** MTT assay show that DNase I did not affect MV4-11 viability. **e**, Confocal images and mean EdU fluorescence intensity quantification demonstrate no significant difference in EV-DNA uptake by BM-MSCs when MV4-11 sEVs with and without DNase I were used.

a

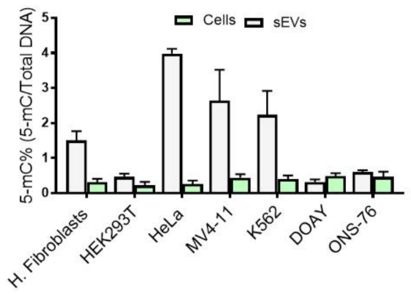

b

②

UniProt n°	EV-chromatin proteins	Expression level
P63261	Actin, cytoplasmic 2	17.88 ± 1.24
O75342	Arachidonate 12 lipoxygenase 12R type	18.675 ± 1.30
A6NMY6	Putative annexin A2-like protein	21.55 ± 0.77
A6NMY6	Annexin A2	21.65 ± 0.91
P05089	Arginase-1	21.45 ± 1.34
P25705	ATP synthase subunit alpha	21.89 ± 1.99
Q13867	Bleomycin hydrolase	20.95 ± 0.77
P31944	Caspase-14	22 ± 1.41
P31944	Caspase-14 subunit p17	22 ± 1.41
P04040	Catalase	16.75 ± 1.06
P81605	Dermcidin-1	23.075 ± 0.81
Q02413	Desmoglein-1	21.95 ± 0.63
P15924	Desmoplakin	21.025 ± 1.37
P68104	Elongation factor 1-alpha	16.25 ± 1.06
P68104	Elongation factor 1-alpha like 3	16.25 ± 1.06
P06733	Alpha-enolase	18.365 ± 0.89
Q01469	Fatty acid binding protein, epidermal	21.25 ± 1.06
P04406	GAPDH	19 ±1.41
P68871	Hemoglobin subunit beta	21.5 ± 0.70
P68871	LVV-hemorphin-7	21.5 ± 0.70
P68871	Spinorphin	21.5 ± 0.70
P14923	Junction plakoglobin	21.875 ± 0.17
Q5T749	Keratinocyte proline-rich protein	21.875 ± 1.23
Q9HC84	Mucin-5B	16.5 ± 0.77
Q13835	Plakophilin-1	18.2 ± 1.18
P29508	Serpin B3	17.65 ± 0.92
P22758	Protein glutamine gamma glutamyltransferase K	18.95 ± 0.62
Q08188	Protein glutamine gamma glutamyltransferase E	17.84 ± 1.18

c

③

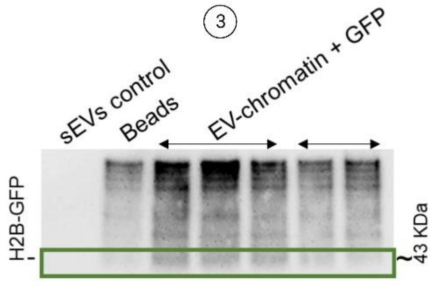

Supplementary Fig. S3. Characterization of chromatin-like in sEVs. a, Methylation profile of DNA derived from the studied sEVs and cell lines. Data are expressed as mean ± SD. **b**, Mass Spec analysis of the pulled-down chromatin (with anti-dsDNA), showing EV-Chromatin related proteins. Data are expressed as mean ± SD. **c**, Western blotting against GFP demonstrating the presence of the fused H2B-GFP protein in EV-Chromatin after pull down using anti-dsDNA.

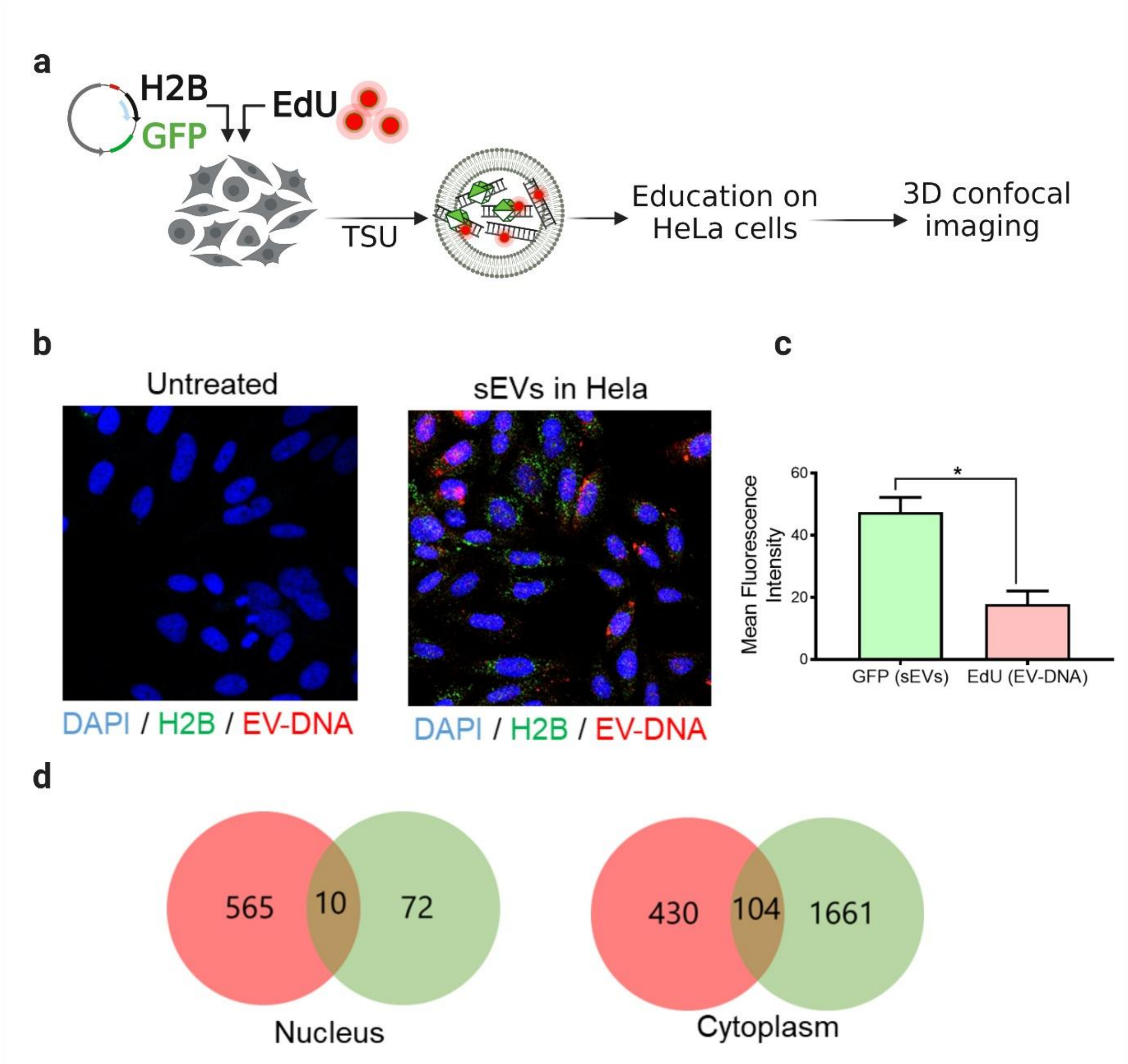

Supplementary Fig. S4. EV-DNA and EV-chromatin uptake by recipient cells. **a**, Schematic illustration showing the education of HeLa cells with EVs derived from HEK293T cells transfected with H2B-GFP plasmid and labeled with EdU. **b**, 2D confocal images showing the uptake of EV-DNA (red) and H2B (green) by the recipient cells. **c**, Quantification of red and green signals in cells treated with labeled sEVs. Data are mean ± SD. **d**, Venn diagram showing the proportion of EV-DNA and histone H2B co-localization in both cytoplasm and nucleus determined by 3D analysing of the z stack confocal images using Imaris software.

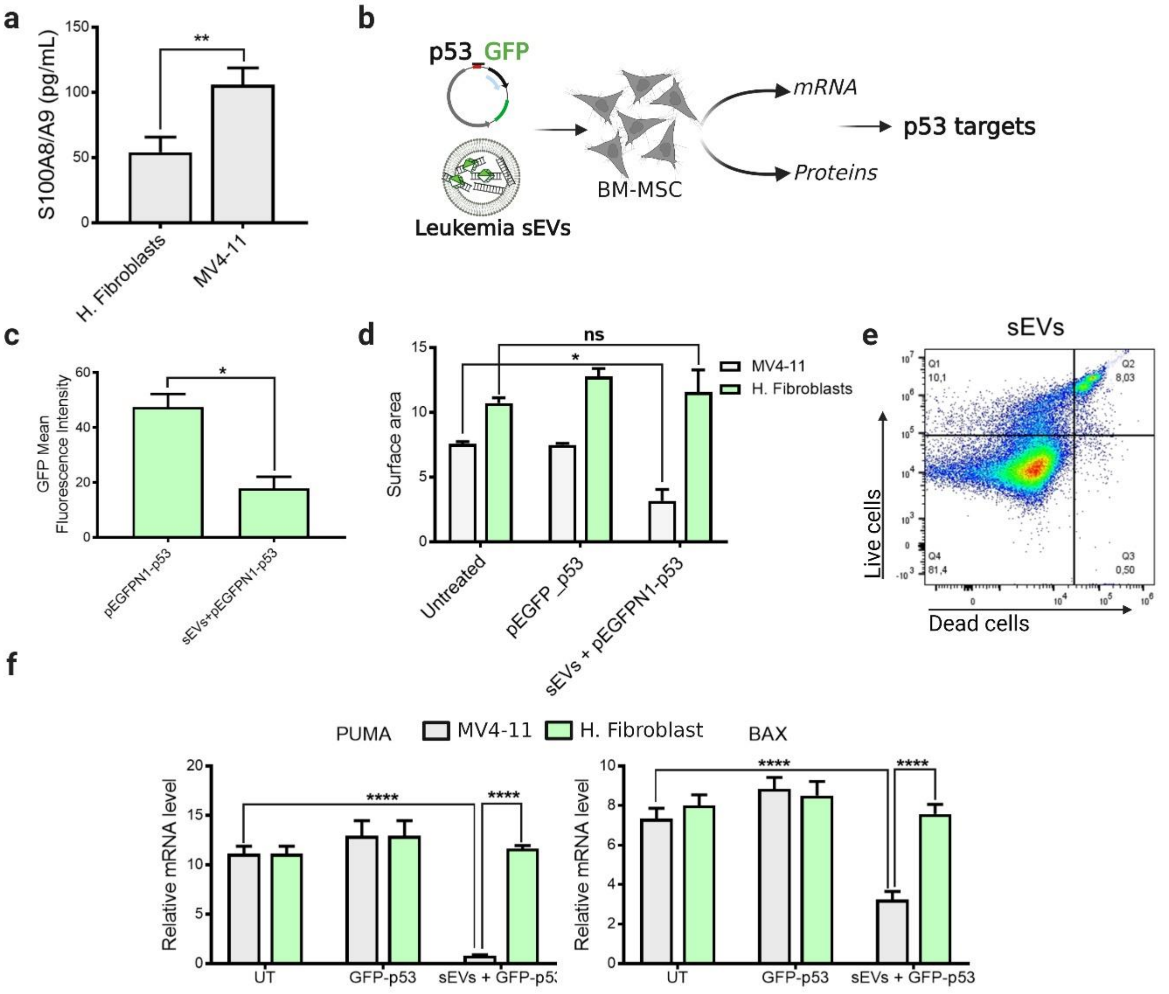

Supplementary Fig. S5. Leukemia sEVs downregulate p53-target genes expression but not MDM2 expression. **a**, S100A8/A9 complex content in sEVs derived from MV4-11 and human fibroblasts cell lines. Data are mean ± SD. **b**, Schematic illustration of the working flow. **c**, GFP fluorescence decreases upon treatment of BM-MSCs with leukemic sEVs, indicating a possible p53 degradation. Data are mean ± SD. **d**, Western blotting band intensity of p53-GFP measured by Image J, revealing a decrease in p53 level upon treatment with leukemic sEVs. Data are mean ± SD. **e**, Annexin V-FITC/ PI staining of BM-MSCs after treatment with leukemic sEVs. **f**, Downregulation of p53 target genes PUMA and BAX after treatment of BM-MSC by MV4-11 sEVs but not H. fibroblast sEVs. Data are mean ± SD, the p-value was calculated using unpaired *t*-test or two-way ANOVA followed by Tukey's multiple comparison test. * p < 0.1; **** p < 0.0001; ns, not significant.

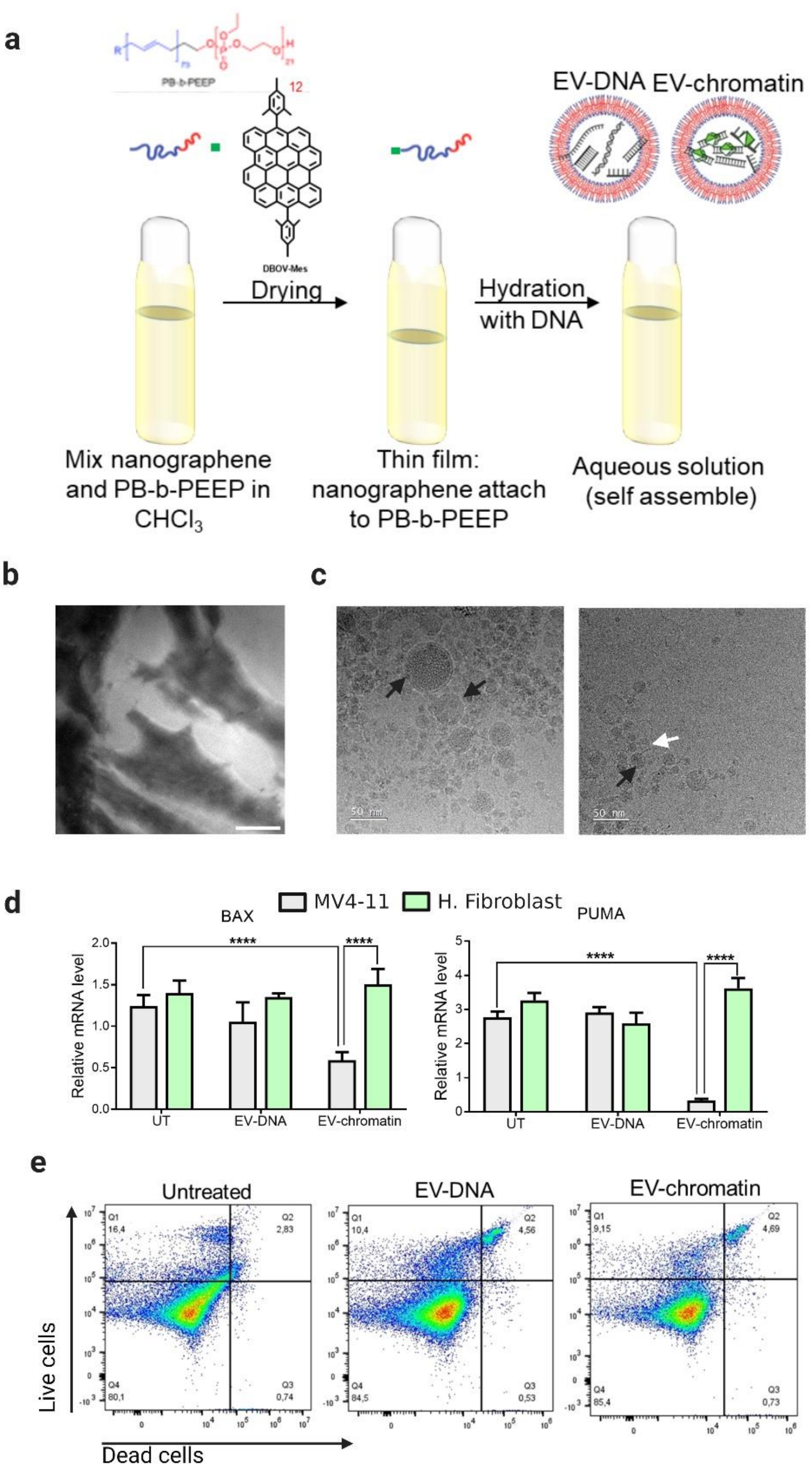

Supplementary Fig. S6. EV-chromatin, but not EV-DNA, promotes BM-MSC proliferation. **a**, Polymersomal formulation of EV-DNA and EV-Chromatin. **b**, wide field images showing the polymersomes uptake. Scale bars- 10 µm. **c**, Cryo-EM images documenting polymersomes bilayer structure (black arrows) and EV-DNA filaments (white arrows) on the surface. Scale bars- 50 nm. **d**, Relative expression levels of p53-target genes PUMA and BAX in BM-MSCs (normalized to the internal control). Human Fibroblasts derived EV-chromatin had no significant effect on BAX and PUMA expression. Data are mean ± SD. **e**, Representative Flow cytometry plots showing the effect of sEVs, EV-DNA, and EV-chromatin treatment on BM-MCSs viability. p-value was calculated by two-way ANOVA followed by Tukey’s multiple comparison test. **** $p < 0.0001$.

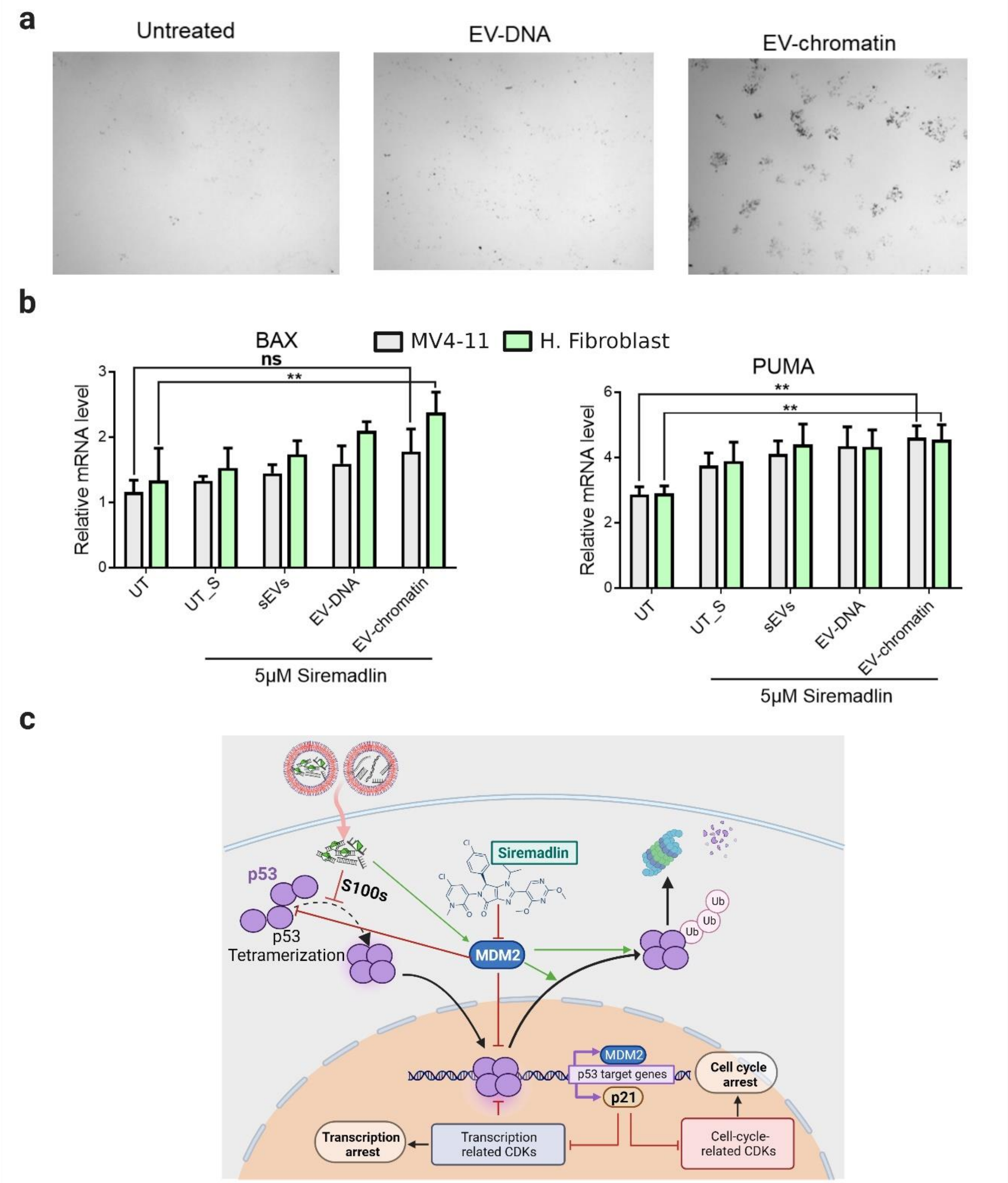

Supplementary Fig. S7. Siremadlin reconstitutes the p53 activity in BM-MSC. **a**, BM-MSC colonies imaging after 7 days. **b**, Relative expression level of apoptotic genes BAX and PUMA after treatment with 5 µM of Siremadlin. Data are mean ± SD. p-value was calculated by two-way ANOVA followed by Tukey's multiple comparison test. $p < 0.01$; ns, not significant. **c**, Possible mechanism by which EV-chromatin promote BM-MSC proliferation. S100 proteins derived from EV-chromatin prevent the tetramerization of p53, which leads to its cytoplasmic accumulation and MDM2-mediated ubiquitination and degradation.

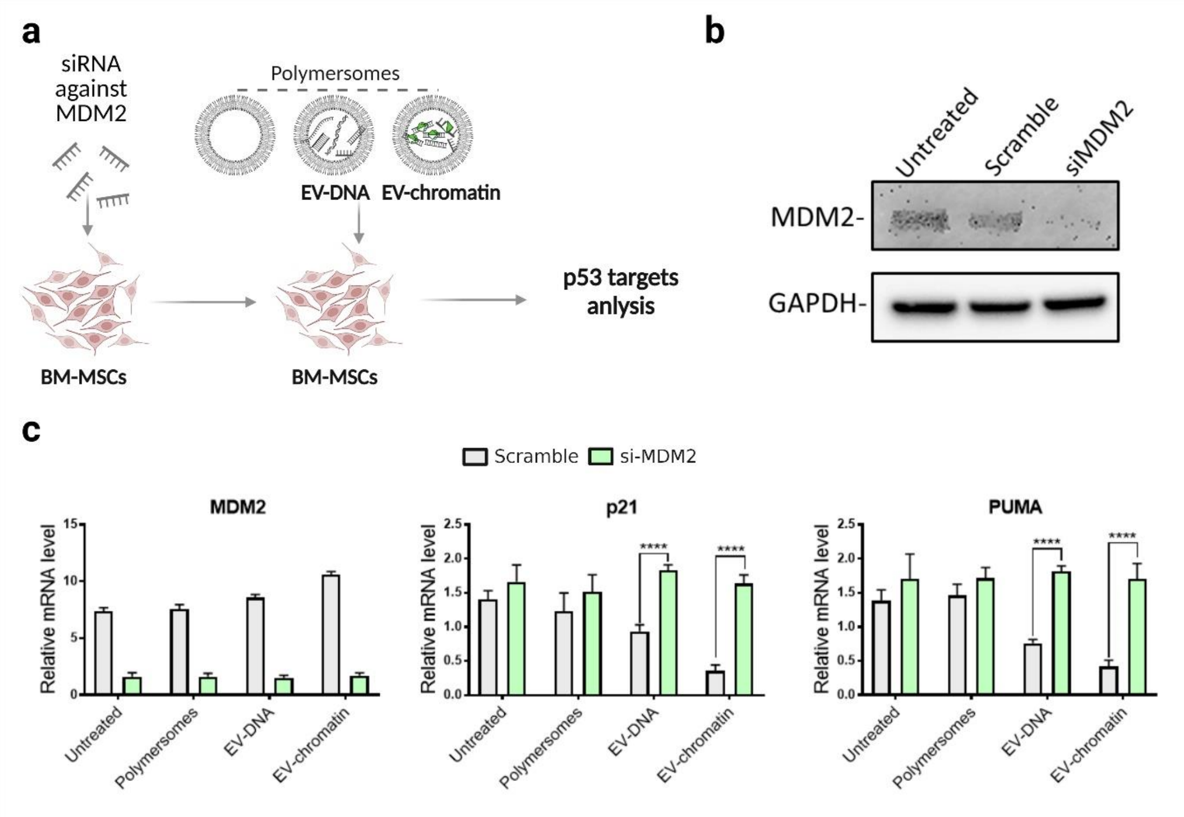

Supplementary Fig. S8. MDM2 knockdown rescues the p53 activity in BM-MSC. **a**, Work flow. **b**, Immunoblots showing the efficiency of MDM2 gene knockdown. **c**, Graphs showing the relative expression level of p53 target genes MDM2, p21, and PUMA with and without si-MDM2. Data are represented as mean ± SD. p-value was calculated by two-way ANOVA followed by Tukey's multiple comparison test. **** $p < 0.0001$.

Supplementary Table S1. Antibodies used in this study

Antibody	Source	Catalogue Number	Assay
Anti-Histone H3	Cell Signalling technology	4499T	WB
Anti-Histone H2B	Abcam Biotechnology	ab1790	WB
Anti-Histone H3 (trimethyl K9)	Abcam Biotechnology	Ab8898	WB
Anti-Histone H4	Cell Signalling technology	2935T	WB
Anti-TSG101	Sigma	HPA006161	WB
Anti-Hsp70	System Biosciences	EXOAB-KIT-1	WB
Anti-CD81	Biolegend	349502	WB
Anti-Syntenin	Abcam Biotechnology	ab133267	WB
Anti-Calnexin	Abcam Biotechnology	ab22595	WB
Anti-LC3B	Sigma	L7543	WB
Anti-dsDNA	Abcam Biotechnology	Ab27156	ChIP
Anti-CD81-FITC	Beckman Coulter	B25329	FC
Anti-p53	Cell Signalling technology	2524S	WB
Anti-GFP	Cell Signalling technology	2555S	WB
Anti-MDM2	Merckmillipore	OP46	WB
Anti-GAPDH	Santa Cruz Biotechnology	sc-47724	WB

FC, flow cytometry; WB, Western blotting; ChIP, chromatin immunoprecipitation.

Supplementary Table S2. Primers for human mRNA expression analysis

Gene	Forward primer	Reverse primer
p21	ATGTGTCCTGGTTCCCGTCCT	CATTGTGGGAGGAGCTGTGA
MDM2	TTCAGTGGGCAGGTTGACTC	CCAGCTGGAGACAAGTCAGG
BAX	GTGTCTCAAGCGCATCGGGGAC	GAGGAGTCTCACCCAACCACCCTG
PUMA	GACGACCTCAACGCACAGTA	CTGGGTAAGGGCAGGAGTC
Bcl-2	ATGTGTGTGGAGAGCGTCAA	ACAGTTCCACAAAGGCATCC
CXCL12	TGGGCTCCTACTGTAAGGGTT	TTGACCCGAAGCTAAAGTGG
SCF	AATCCTCTCGTCAAAACTGAAGG	CCATCTCGCTTATCCAACAATGA
COL1A1	GTTGAGTTTGGGTTGCTTGTC	CCTGTCTGCTTCCTGTAAACT
ANGPT1	GCCATCTCCGACTTCATGTT	CTGCAGAGAGATGCTCCACA
GAPDH	AATCCCATCACCATCTTCCA	TGGACTCCACGACGTACTCA
